# Acoustic phonon modes and phononic bandgaps in GaN/AlN nanowire superlattices

**DOI:** 10.1186/1556-276X-7-479

**Published:** 2012-08-23

**Authors:** Seiji Mizuno

**Affiliations:** 1 Department of Applied Physics, Hokkaido University, Kita 13 Nishi 8, Kita-ku, Sapporo, 060-8628, Japan

**Keywords:** Nanowire superlattice, Acoustic phonon, Phononic crystal, Phononic bandgap

## Abstract

We study numerically the phonon dispersion relations and corresponding displacement fields for a circular cross-section nanowire superlattices consisting of anisotropic GaN and AlN. We determine a set of parameters which gives complete phononic bandgaps. The results suggest the potential for manipulating phonons in the micro/nano electromechanical systems.

## Background

Phononic crystals (PCs) are composite materials made of arrays of constituents embedded in host materials [1-3]. The interesting characteristics of the PCs are related to the existence of phononic bandgaps (i.e., frequency gaps) due to the Bragg reflections of the phonons with long wavelengths. We can regard the PC as an opaque barrier for the phonons within the phononic bandgaps [4]. This suggests the potential for designing various phonon optic devices, such as phonon filters, mirrors, resonators, etc.

Recent advances in fabrication methods enable realization of one-dimensional hetero-structures, i.e., nanowire superlattices (NWSLs) [5-10]. Their electronic and optical properties were studied, and a variety of possible applications utilizing the characteristics were also proposed [11-14]. In addition, the NWSLs are expected to yield interesting physical effects on phonons, which influence the electronic states and the transport properties via the electron-phonon interaction. These NWSLs can be regarded as wire-type phononic crystals (WPCs), in which the phononic bandgaps are induced by the periodicity along the wire axis.

In a previous paper [15], we developed a numerical method to derive phonon modes in a free-standing NWSL of anisotropic material with an arbitrary shape of cross-section. As examples, the phonon modes were calculated for the rectangular and square cross-section GaAs/AlAs and InP/InAs NWSLs composed of anisotropic materials [15,16].

Though above result revealed the important aspects of phonon modes in the NWSLs, it seems to be difficult to design WPCs with complete phononic bandgaps because in the dispersion relations of these NWSLs, many subbands are folded into the mini-Brillouin zone and the frequencies of gaps are different with phonon modes. In addition, the gap widths are narrow in these NWSLs because the difference of the acoustic impedance between the GaAs and AlAs layers (or between the InP and InAs layers) is small. The NWSLs with large acoustic mismatch would be suitable for designing the phonon optic devices.

In the present work, we numerically calculate the dispersion relations and corresponding displacement fields for a circular cross-section NWSL consisting of GaN and AlN, and we determine a set of parameters which gives complete frequency gaps.

## Methods

The equation giving the eigenfrequencies of phonon modes in a freestanding NWSL composed of anisotropic crystals was formulated in [15]. In this method, the displacement components u_i_ (*i* = *x,y,z*) are expanded in terms of a set of basis functions *ϕ*_*α*_ (**r**)

(1) $$u_{i}(r)=\underset{\alpha }{\sum }A_{\mathrm{\alpha i}}\phi_{\alpha }(r).$$

The expansion coefficients *A*_*αi*_ and the eigenfrequencies *ω* are determined by solving the generalized eigenvalue equation:

(2) $$\underset{\alpha ,\ell }{\sum }H_{\mathrm{\beta i},\alpha \ell }A_{\alpha \ell }=\omega^{2}\underset{\alpha ,\ell }{\sum }S_{\mathrm{\beta i},\alpha \ell }A_{\alpha \ell }.$$

For the NWSLs composed of cubic materials, the matrix elements *H*_*βi*,*αℓ*_ and *S*_*βi*,*αℓ*_ are written as

(3) $$\begin{array}{ll} H_{\beta i,\alpha \ell } & =\left\{\langle i\beta \left|C_{11}\right|i\alpha \rangle +\underset{j\neq i}{\sum }\langle j\beta \left|C_{44}\right|j\alpha \rangle \right\}\delta_{i\ell }+\left\{\langle i\beta \left|C_{12}\right|\ell \alpha \rangle \right)\\ & \;\left(+\langle \ell \beta \left|C_{44}\right|i\alpha \rangle \right\}\left(1-\delta_{i\ell }\right), \end{array}$$

(4) $$S_{\beta i,\alpha \ell }=\delta_{i\ell }\langle \beta \left|\rho \right|\alpha \rangle ,$$

where

(5) $$\langle j\beta \left|C_{\mu \nu }\right|k\alpha \rangle =\underset{V}{\int }\frac{\partial \phi_{\beta }^{*}(r)}{\partial x_{j}}C_{\mu \nu }(r)\frac{\partial \phi_{\alpha }(r)}{\partial x_{k}}dr,$$

(6) $$\langle \beta \left|\rho \right|\alpha \rangle =\underset{V}{\int }\phi_{\beta }^{*}(r)\rho (r)\phi_{\alpha }(r)dr.$$

Here, *C*_*μν*_and *ρ* are the stiffness tensor and mass density, respectively, which are dependent on **r** in the NWSLs.

As basis functions, we adopt the product of powers of the Cartesian coordinates in the *xy* plane. The *z* dependence is expressed in the form of the Bloch wave:

(7) $$\phi_{\alpha }(k,r)=\frac{1}{\sqrt{V}}{\left(\frac{x}{R}\right)}^{m}{\left(\frac{y}{R}\right)}^{n}e^{i\left(k+G\right)z}.$$

Here, *R* denotes the radius of the wire; *G* is the reciprocal lattice vector; and *V* = *ΠR*^2^*D* is the volume of the unit cell, where *D* is the length of the unit cell in the *z* direction. The basis functions are specified with *α* = (*m*,*n*,*G*). The expressions for the matrix elements *H*_*βi*,*αℓ*_and *S*_*βi*,*αℓ*_ can be analytically obtained for the circular NWSLs.

Based on group theory [17,18], the phonon modes are classified symmetrically. In the present study, the constituent layers are assumed to be cubic materials, i.e., zinc-blende structure. The group of *k* is *C*_4*v*_ for 0 < *k* < *Π*/*D*. The irreducible representations are A_1_, A_2_, B_1_, B_2_, and E [15].

By considering the above symmetry, the symmetry-adapted basis function in the present system can be constructed, and the phonon dispersion relations of each mode are independently calculated.

## Results and discussion

The boundary condition at the free surface of the wire requires that the wave numbers in the lateral direction are discretized. On the other hand, the wave number *k* in the longitudinal direction has a continuous value. Therefore, even the homogeneous plain nanowire has subband structure. In the NWSL, the subbands are folded into the mini-Brillouin zone. In other words, the size of the mini-Brillouin zone and corresponding phononic bandgaps are determined by the periodicity *D* of the NWSL.

For the nanowires with smaller *R*, the maximum wavelengths in the lateral directions become shorter. As a result, the subbands except for the lowest dispersion curve of each mode go up to the higher frequency region, though a lot of subbands are folded into the mini-Brillouin zone. Changing the ratio of *R* and *D*, we can control the phonon modes in the lower frequency range.

**Figure 1:**
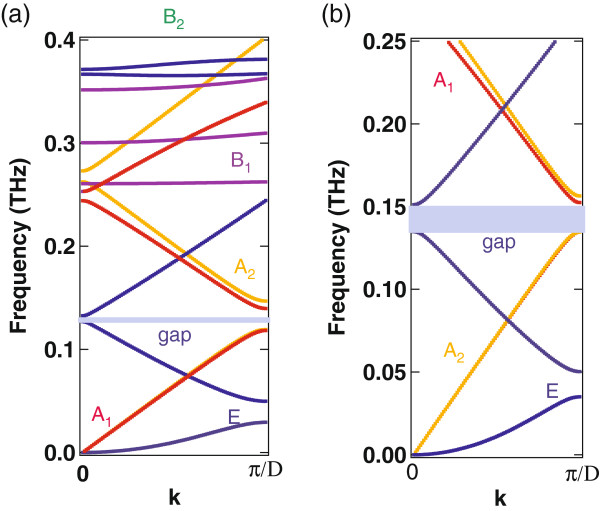
**Phonon dispersion relations of the circular cross-section GaN/AlN NWSL.** (**a**) *R* = 5.0 nm and *D* = 20.0 nm; (**b**) *R* = 5.0 nm and *D* = 45.0 nm (thicknesses of GaN and AlN layers are 25.0 and 20.0 nm, respectively). The B_2_ modes exist in higher frequency range, and all the E modes are doubly degenerated.

Figure 1a illustrates the phonon dispersion relations calculated for *R* = 5.0 nm and *D* = 20.0 nm (thicknesses of both GaN and AlN layers are 10.0 nm). Other parameters we used are as follows: *ρ*= 6.15 g/cm^3^, C_11_ = 2.96, C_12_ = 1.54, and C_44_ = 2.06 (all in units of 10^12^ dyn/cm^2^) for GaN; *ρ* = 3.26 g/cm^3^, C_11_ = 3.04, C_12_ = 1.52, and C_44_ = 1.99 (all in units of 10^12^ dyn/cm^2^) for AlN [19].

In the present frequency range, we can see four different modes, i.e., the A_1_, A_2_, B_1_, and E modes. The dispersion curves corresponding to the B_2_ modes exist in higher frequency range. The lowest dispersion curve of the A_2_ mode is mostly overlapped with that of the A_1_ mode.

The complete frequency gaps are realized for the parameters we selected. For comparison, we show in Figure 1b the phonon dispersion relations calculated for *R* = 5.0 nm and *D* = 45.0 nm (thicknesses of GaN and AlN layers are 25.0 and 20.0 nm, respectively). In this example, the broader bandgaps of the A_1_, A_2_, and E modes are nearly coincident with each other. Here, we note that the complete bandgaps disappear if the isotropic approximation is used for each constituent layer.

The lowest dispersion curves of the A_1_ and A_2_ modes are linear in *k*, i.e., *ω* vanishes at *k* = 0. On the other hand, the lowest frequencies of the B_1_ and B_2_ modes have finite values. For thin (thick) NWSLs, the lowest frequencies of B_1_ and B_2_ modes become higher (lower).

**Figure 2:**
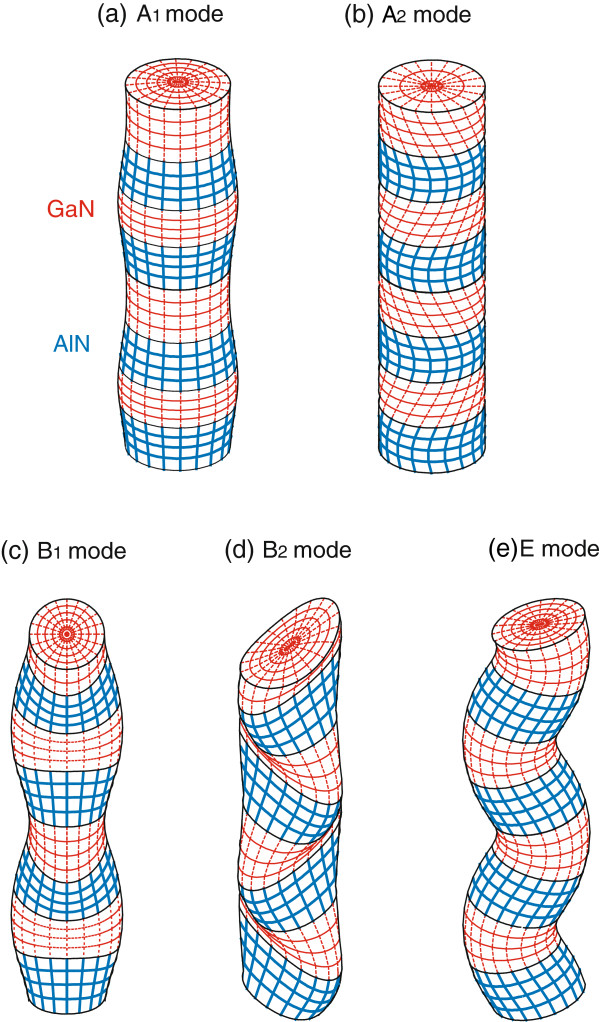
**Displacement field patterns corresponding to the lowest (a) A_1_,** (**b**) **A_2_,** (**c**) **B_1_,** (**d**) **B_2_, and** (**e**) **E modes at*k*=*Π*/*D*.**

Figure 2a,b shows the displacement patterns corresponding to the lowest A_1_ and A_2_ modes at *k* = *Π* / *D*, respectively. The lowest A_1_ mode has the feature of a dilatational mode, while the lowest A_2_ mode shows the feature of a torsional mode.

Figure 2c,d,e shows the displacement patterns corresponding to the B_1_, B_2_, and E modes at *k* = *Π* / *D*, respectively. The lowest B_1_ mode shows the feature of a stretching mode, i.e., the alternating dilatation and contraction in the *x* and *y* directions, while the lowest B_2_ mode shows the features of a shear mode, i.e., alternating stretching in the two diagonal directions.

All dispersion curves of the E mode are doubly degenerate because the irreducible representation of the E mode is two-dimensional. For the E modes, the lowest dispersion curve near *k* = 0 is proportional to *k*^2^. This parabolic behavior is due to the fact that these modes correspond to the bending of the NWSL. Figure 2e clearly shows that the E modes have a feature of flexural mode.

## Conclusion

We theoretically studied the acoustic phonon modes in circular cross-section NWSLs consisting of cubic GaN and AlN. We calculated their dispersion relations and phonon displacement fields. These modes are classified into five types, i.e., A_1_, A_2_, B_1_, B_2_, and E modes, which have features of dilatational, torsional, stretching, shear, and flexural modes, respectively. We determined a set of parameters which gives complete phononic bandgaps. The results suggest the realization of the optimized phonon devices, such as phonon filters or mirrors in the micro/nano electromechanical systems.

In the present work, we only showed the results for the NWSLs consisting of cubic materials (i.e., zinc-blende structure). The results for the NWSLs with wurtzite structure will be given and the difference will be discussed elsewhere.

## Competing interests

The author declares that he has no competing interests.
